# The pathogenesis of formal thought disorder – towards an integrative view

**DOI:** 10.1192/j.eurpsy.2023.1296

**Published:** 2023-07-19

**Authors:** E. Dornelles, D. Telles-Correia

**Affiliations:** CLINICA UNIVERSITÁRIA DE PSIQUIATRIA E PSICOLOGIA MÉDICA, FACULDADE MEDICINA UNIVERSIDADE DE LISBOA, Lisboa, Portugal

## Abstract

**Introduction:**

Formal Thought Disorder (FTD) is a cluster of symptoms and signs, and can be summarized as a multidimensional construct, reflecting idiosyncrasies in thought, language, and communication in general. The inquiry into its etiology is complicated by the ambiguity of the construct itself, and many theories regarding its pathogenesis have been put forward. Two main neurocognitive models, however, have been garnering attention in mainstream FTD research: the “dyssemantic” and the “dysexecutive” hypotheses. These concepts have been classically pitted out against each other as mutually exclusive, but recent studies have proposed a more integrative view.

**Objectives:**

In this presentation, we aim to explore the two main models for explaining FTD pathogenesis, and to show how an integrative model which accounts for both the dyssemantic and dysexecutive deficits seen in patients with FTD might be better at explaining its etiology.

**Methods:**

We conducted a systematic review of the available literature according the PRISMA 2020 statement. We began by researching the Pubmed and Cochrane databases using the following search string: ((“Formal thought disorder*”[Title/Abstract]) AND (dysexecutive[Title/Abstract])) OR ((“Formal thought disorder*”[Title/Abstract]) AND (dyssemantic[Title/Abstract])) OR ((“Formal thought disorder*”[Title/Abstract]) AND (pathogenesis[Title/Abstract])) OR ((“Formal thought disorder*”[Title/Abstract]) AND (etiology[Title/Abstract])). 20 articles were retrieved, along with 2 ongoing trials. We screen for a total of 12 included articles. We also included 17 articles from citation searching, resulting in a final count of 29 included articles. We then summarized the main findings.

**Results:**

Two influential hypotheses explaining the neurocognitive pathogenesis of different FTD symptom are the “dyssemantic” and “dysexecutive” hypotheses. The “dyssemantic” model emphasizes abnormalities in language-processing related brain regions and functional networks. Some studies suggest that the dysfunctions might involve higher-order semantics and the syntactic component. The “dysexecutive” hypothesis suggests that impaired planning and monitoring might lead to poorly formulated or prone-to-error speech. Recent studies, however, have suggested that FTD might be related to a combination of both executive dysfunction and impaired semantic processing, which would then combine in different proportions and yield the different FTD manifestations.

**Conclusions:**

While disfunctions in both semantic and executive cognitive faculties have been independently explored as potential explanations for the pathogenesis of FTD, a more integrative picture has surfaced in recent research. It proposes that FTD might actually be the reflections of a combination of different proportions of disfunctions in the executive and/or linguistic processes. More research is needed, with better defined FTD dimensions, in order to further explore this model.

**Disclosure of Interest:**

None Declared

